# Identification of Pyruvate Dehydrogenase E1 as a Potential Target against *Magnaporthe oryzae* through Experimental and Theoretical Investigation

**DOI:** 10.3390/ijms22105163

**Published:** 2021-05-13

**Authors:** Yuejuan Li, Baichun Hu, Zhibin Wang, Jianhua He, Yaoliang Zhang, Jian Wang, Lijie Guan

**Affiliations:** 1 Department of Pharmaceutical and Biological Engineering, Shenyang University of Chemical Technology, Shenyang 110142, China; liyuejuan6991@gmail.com (Y.L.); tutu102629@gmail.com (Z.W.); jianhuaoh@gmail.com (J.H.); 2 Key Laboratory of Structure-Based Drug Design & Discovery, Ministry of Education, Shenyang Pharmaceutical University, Shenyang 110142, China; hubaichun@outlook.com (B.H.); yaoliang_zhang@outlook.com (Y.Z.); 3 Key Laboratory of Intelligent Drug Design and New Drug Discovery of Liaoning Province, Shenyang Pharmaceutical University, Shenyang 110142, China

**Keywords:** isobavachalcone, RNA sequencing, enzyme activity, pyruvate dehydrogenase, homology modeling, molecular dynamics simulation

## Abstract

*Magnaporthe oryzae* (*M. oryzae*) is a typical cause of rice blast in agricultural production. Isobavachalcone (IBC), an active ingredient of *Psoralea corylifolia* L. extract, is an effective fungicide against rice blast. To determine the mechanism of IBC against *M. oryzae*, the effect of IBC on the metabolic pathway of *M. oryzae* was explored by transcriptome profiling. In *M. oryzae*, the expression of pyruvate dehydrogenase E1 (PDHE1), part of the tricarboxylic acid (TCA cycle), was significantly decreased in response to treatment with IBC, which was verified by qPCR and testing of enzyme activity. To further elucidate the interactions between IBC and PDHE1, the 3D structure model of the PDHE1 from *M. oryzae* was established based on homology modeling. The model was utilized to analyze the molecular interactions through molecular docking and molecular dynamics simulation, revealing that IBC has π-π stacking interactions with residue TYR139 and undergoes hydrogen bonding with residue ASP217 of PDHE1. Additionally, the nonpolar residues PHE111, MET174, ILE 187, VAL188, and MET250 form strong hydrophobic interactions with IBC. The above results reveal that PDHE1 is a potential target for antifungal agents, which will be of great significance for guiding the design of new fungicides. This research clarified the mechanism of IBC against *M. oryzae* at the molecular level, which will underpin further studies of the inhibitory mechanism of flavonoids and the discovery of new targets. It also provides theoretical guidance for the field application of IBC.

## 1. Introduction

Rice is one of the most important staple foods globally, feeding more than half of the world’s total population [1]. Rice blast, caused by *Magnaporthe oryzae*, is arguably the most destructive fungal disease of rice [2]. *M. oryzae* infects plants at almost all growth stages and reduces rice yield by 10–35% [3]. It is estimated that the amount of rice destroyed by rice blast disease each year is enough to feed 60 million people [4]. Therefore, effective methods to control this disease are necessary to ensure global food security. Fungicides have been widely used to control fungal diseases, greatly promoting increased crop production and developing agricultural economies [5]. Synthetic fungicides have played a major role in controlling plant diseases for many years, and they are diverse, highly effective, and economical. Despite the advantages of synthetic fungicides, their long-term use has led to problems, such as destruction of the natural balance, environmental pollution [6], residual toxicity, and developing drug resistance. This has prompted intensive research on developing biopesticides, including botanical fungicides [7], as a strategy for developing safer formulations to champion sustainable [6] and eco-friendly agriculture. Such developments are of great significance, especially in the face of a growing global population, climate change, and the increasing resistance of fungi to existing compounds [8].

Studies have found some bioactive compounds can be extracted from the seeds of *Psoralea corylifolia* L., which is a traditional herb with various ethnobotanical applications [9]. The seeds of this plant have potent medicinal activities and can be used in various traditional medicines to treat various diseases [10]. *Psoralea corylifolia* L. has been found to possess significant antifungal, antibacterial, antitumor, antidiabetic [11], and estrogenic effects and can be locally applied for alopecia leukoderma, inflammation, psoriasis, leprosy, and eczema [9]. It was found that an alcoholic extract of *P. corylifolia* seed can stimulate the immune system in mice by increasing cell-mediated and humoral immune responses [12]. In addition, other results have indicated that *P. corylifolia* seed extract also inhibits the production of ROS in HepG2 cells and hepatocytes of aged mice, in addition to enhancing superoxide dismutase activity [13]. Studies have found that the active compounds bakuchiol, psoralidin, bavachin and isobavachalcone [10] can effectively inhibit fungi. Isobavachalcone (IBC), a flavonoid compound used in agriculture as a botanical fungicide, is the major active compound in *Psoralea corylifolia* L. extract. We found that IBC has special inhibitory effects against many pathogenic microorganisms, such as *M. oryzae*, *Valsa mali*, *Fusarium graminearum*, *Colletotrichum orbiculare*, and *Colletotrichum coccodes*, the inhibition rate of spore germination by IBC for *M. oryzae* reached 98%. Agar diffusion experiments showed that the crude extraction had a notable inhibitory effect on *M. oryzae*, with an EC_50_ value of 40.550 mg/L. The current research on fungicide mechanisms has mainly focused on the influence of cell wall synthesis and degradation, cell membrane permeability and integrity, signal transduction, energy metabolism, total bacterial protein, and nucleic acid. Research has demonstrated that the *V. mali* mycelium was damaged, and the mitochondrial membrane, cell membrane, and nuclear membrane boundaries became unclear following treatment with IBC. In addition, the cytoplasm material was obviously condensed and exosmosed, and the volume of vacuoles in the cells increased significantly [14].

The pyruvate dehydrogenase complex (PDHc) is an organized multi-enzyme complex held together by noncovalent bonds, which catalyzes a continuous reaction [15]. PDHc is located on the mitochondrial membrane. As one of the key enzymes in the central metabolic system, PDHc plays an important role in cellular metabolism by catalyzing the oxidative decarboxylation of pyruvate to acetyl-CoA, a key step in the production of cellular energy [16,17]. Therefore, the PDHc has been reported as a potential target for agrochemical design and antimicrobial agents [18,19]. Builie found that alkylacylphosphonates (salts) are lethal to plants by inhibiting PDHc [20], which indicated that PDHc could be used as a target for herbicides. He Hongwu’s research group [19] created a PDHc inhibitor herbicide (Clacyfos, HW02) with high efficiency, low toxicity, and good environmental compatibility that is low residue. This research confirmed the rationality and feasibility of designing high-efficiency and high selectivity herbicide molecules for targeting PDHc.

As a new plant-derived fungicide, IBC was used for agricultural disease control for the first time. The inhibitory mechanism of IBC has not been studied in-depth. In our previous research, we analyzed the mechanism of IBC acting on the fungal wall and membrane, which revealed that IBC could bind chitinase in *M. oryzae*; this led to activation of the hydrolyzing ability of the chitinase of *M. oryzae* and structural damage of the cell wall. This work revealed the inhibitory mechanism of IBC at the molecular level to allow the discovery of new targets of IBC, which will help develop new and effective fungicides toward overcoming problems of resistance.

To explore the mechanism of IBC against *M. oryzae*, the rice blast fungus was treated with IBC for transcriptome profiling and enzyme activity determination. This research focused on discovering IBC’s effect on pyruvate dehydrogenase E1. At the same time, the interaction between IBC and pyruvate dehydrogenase E1 was studied by homology modeling, active site recognition, molecular dynamics, and molecular docking techniques. Through the research results, the binding mode of IBC and pyruvate dehydrogenase E1 was studied, and the interaction between the compound and the target was further explored.

## 2. Results

### 2.1. Analysis of Differentially Expressed Genes

**Figure 1:**
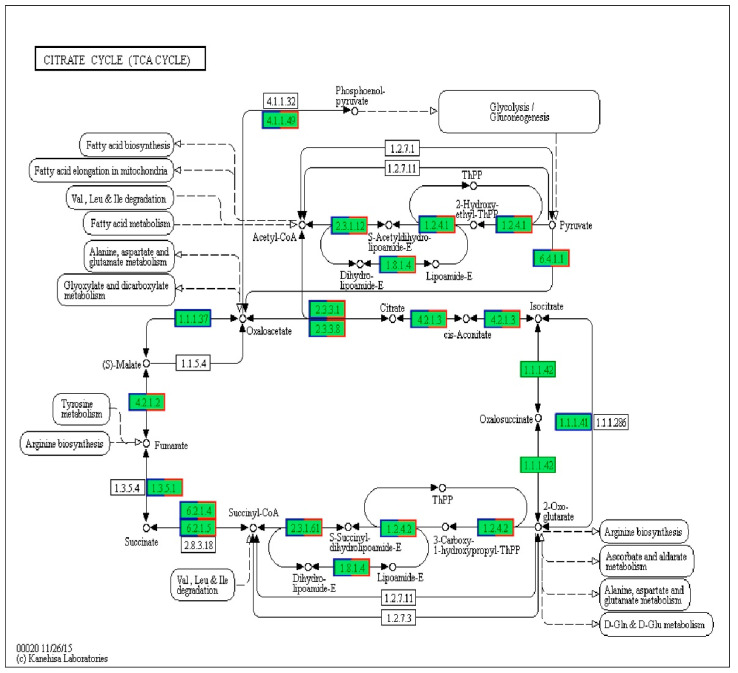
Pathway of the TCA cycle. The unigenes annotated by KEGG are marked with a green background. Blue and red borders mark DEGs.

According to transcriptome profiling, 1047 differentially expressed genes (DEGs) were enriched in different pathways after treatment with IBC. The signaling pathways involved in the differential genes were reflected by KEGG annotation and enrichment analysis. We found that 14 DEGs in the TCA cycle pathway were changed (Figure 1). The TCA cycle is a key metabolic pathway linking carbohydrate, fat, and protein metabolism. In the whole process, the oxidative decarboxylation of pyruvate generates acetyl-CoA, and acetyl-CoA enters the tricarboxylic acid cycle. The pyruvate dehydrogenase complex and α-ketoglutarate dehydrogenase complex are reductase systems with a catalytic role, and their expression levels were significantly downregulated (Table 1). Six differentially expressed genes of the pyruvate dehydrogenase E1 component, pyruvate dehydrogenase E2 component, dihydrolipoamide dehydrogenase, 2-oxoglutarate dehydrogenase E1 component, 2-oxoglutarate dehydrogenase E2 component, pyruvate carboxylase, and two unchanged genes of isocitrate dehydrogenase (NAD+) and isocitrate dehydrogenase were selected for qPCR verification.

### 2.2. Validation of Differentially Expressed Genes by qPCR

**Figure 2:**
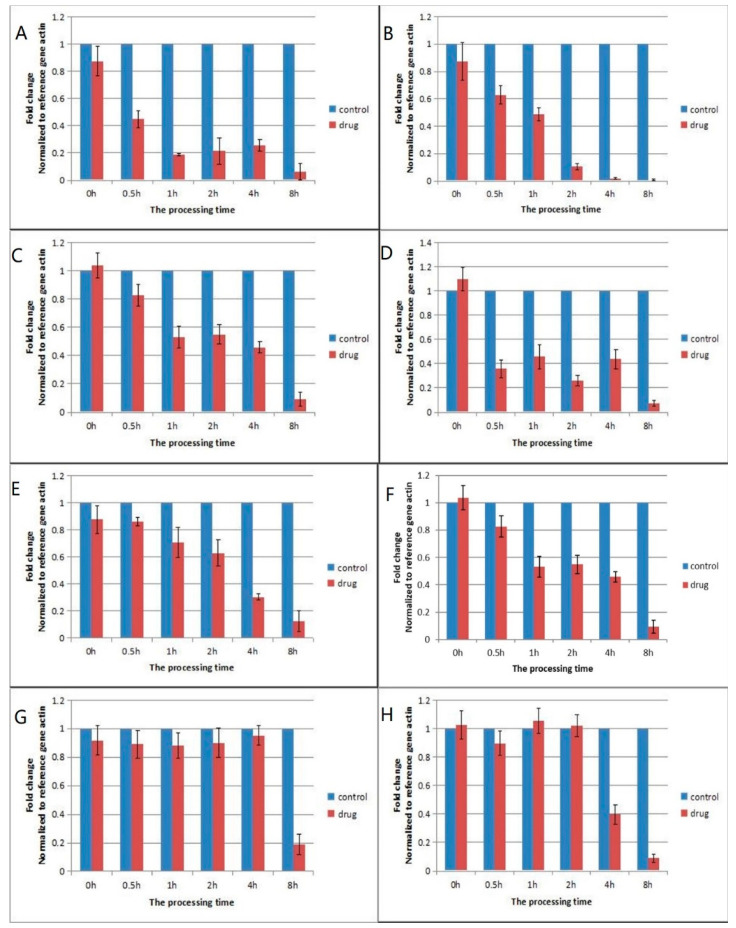
Gene expression at different time points following treatment with IBC for (**A**) pyruvate dehydrogenase E1 component, (**B**) pyruvate dehydrogenase E2 component, (**C**) dihydrolipoamide dehydrogenase, (**D**) 2-oxoglutarate dehydrogenase E1 component, (**E**) 2-oxoglutarate dehydrogenase E2 component, (**F**) pyruvate carboxylase, (**G**) isocitrate dehydrogenase, and (**H**) isocitrate dehydrogenase (NAD+). The x-coordinate is the processing time, and the y-coordinate is the fold change normalized to the reference gene actin.

The results of the qPCR show that the expression of the pyruvate dehydrogenase E1 component decreased to 0.42 after IBC treatment for 0.5 h, then continued declining during the 1–8 h period. The E1 component was the most downregulated compared with the other 5 genes (Figure 2). The expression of isocitrate dehydrogenase and isocitrate dehydrogenase (NAD+) did not change at 2 h after IBC treatment, while the other 6 genes were all downregulated, which was consistent with the results of the RNA-Seq.

### 2.3. Enzymatic ActivityThe

**Figure 3:**
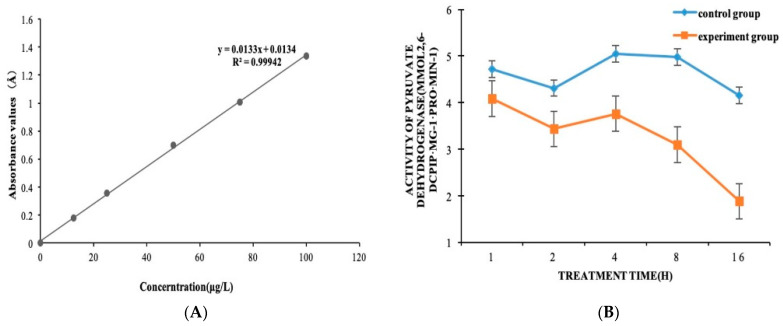
(**A**) Standard curves of 2,6-DCPIP. (**B**) The enzymatic activity of PDH within 16 h of treatment with isobavachalcone and DMSO.

We further studied the effect of IBC on the activity of the pyruvate dehydrogenase E1 component in *M. oryzae* according to the results of RNA-Seq and qPCR. The results showed the enzyme activity of pyruvate dehydrogenase in the experimental groups was 0.87, 0.8, 0.74, 0.62, and 0.45 times as much as in the control group at 1, 2, 4, 8, and 16 h, respectively (Figure 3). It can be seen that the enzyme activity in the cells gradually decreased from 1 h after drug treatment, and the mycelium gradually collapsed and died, causing the TCA cycle pathway of the intracellular substance metabolism hub to be blocked.

### 2.4. Homology Modeling and Prediction of the Active Site of Pyruvate Dehydrogenase

**Figure 4:**
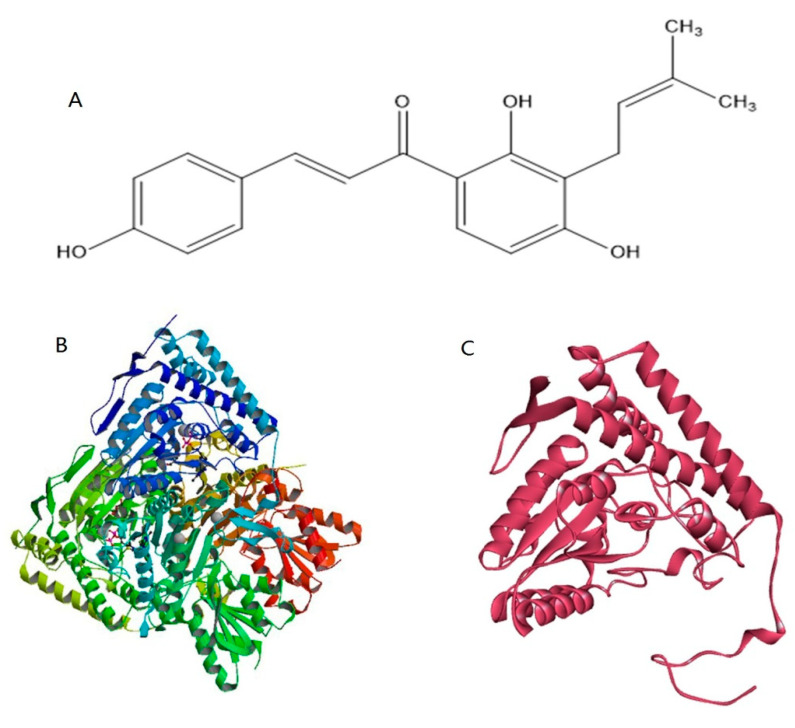
(**A**) Chemical structure of isobavachalcone obtained by Marvin Sketch. (**B**) The schematic diagram of the template protein (PDB code: 3exe). (**C**) The final crystal structure of pyruvate dehydrogenase E1. It was constructed using chain A of G pyruvate dehydrogenase (E1p) component of the human pyruvate dehydrogenase complex as a template.

**Figure 5:**
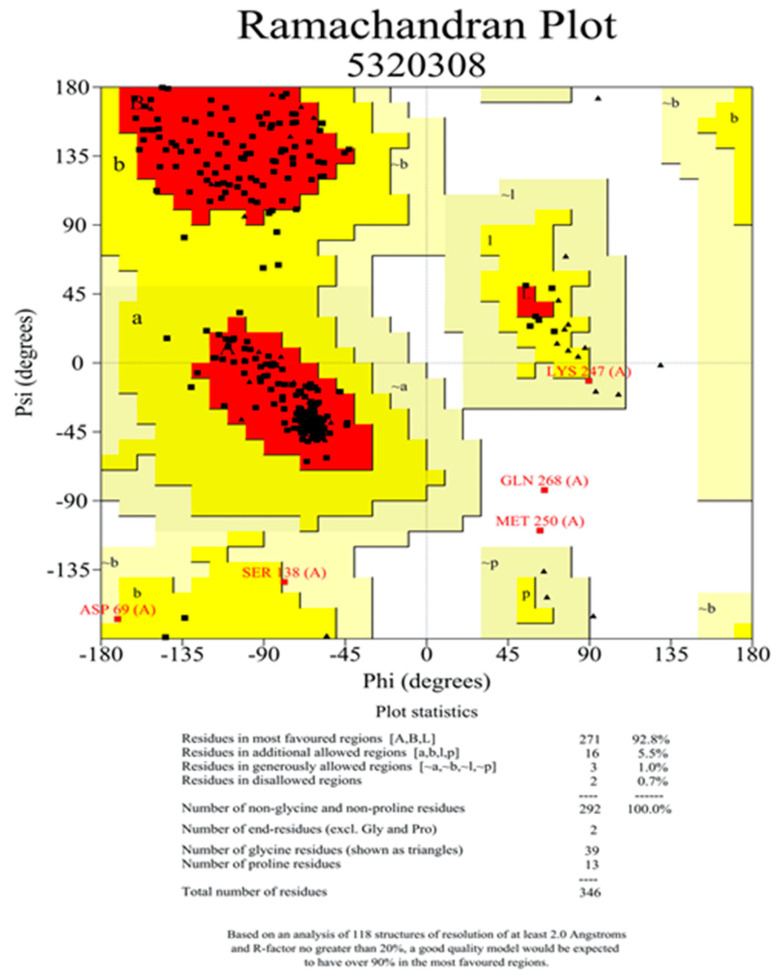
Validation of the homology model of pyruvate dehydrogenase. Ramachandran plots were obtained by Procheck on the website. The Ramachandran plot of the angles of phi and psi showed that the total number of residues was 346, with 92.8% of the residues in the most favorable region (271 residues).

**Figure 6:**
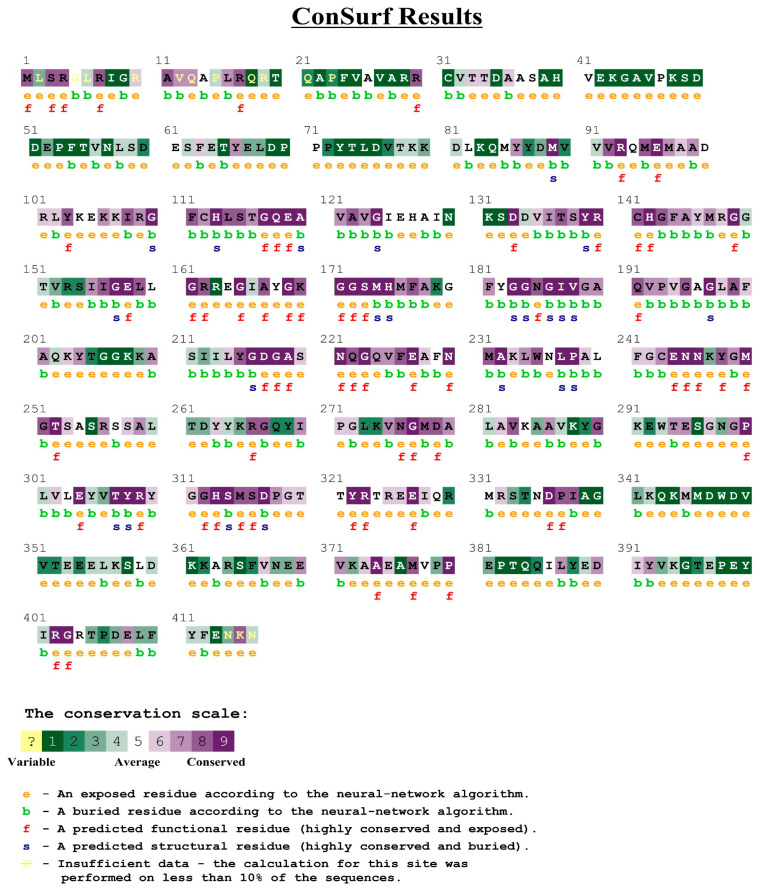
Prediction of the theoretically active site of the pyruvate dehydrogenase E1 receptor. Highly conserved functional residues are enriched in regions 111 dynamics simulation, revealing that IBC has 121, 161–181, 211–231, 241–251 and 311–321.

To obtain the crystal structure of the pyruvate dehydrogenase E1 component of *M. oryzae*, the template protein 3exe, which had high sequence identity with the pyruvate dehydrogenase E1 component of *M. oryzae*, was used for homology modeling. The 3D structure of the template protein is shown in Figure 4B. According to the homology modeling results based on the SWISS-MODEL online server, the identity of the amino acid sequence alignment between the template protein and pyruvate dehydrogenase is 55.13% (Figure S1). A 3D model of pyruvate dehydrogenase was constructed by homology modeling with optimization, and the final result of the modeling is shown in Figure 4C. The Ramachandran plot of the angles of phi and psi showed that the total number of residues was 346, indicating 92.8% of the residues were in the most favorable region (271 residues), while 6.5% were in the allowed region, and two residues were in the not allowed region (Figure 5). The evaluation results indicated that the constructed pyruvate dehydrogenase homology model was reasonable. Meanwhile, the online server ConSurf was used to predict the theoretically active site of the pyruvate dehydrogenase E1 receptor. The results indicated that highly conserved functional residues are enriched in regions 111–121, 161–181, 211–231, 241–251, and 311–321 (Figure 6).

### 2.5. Protein-Ligand Docking Analysis

**Figure 7:**
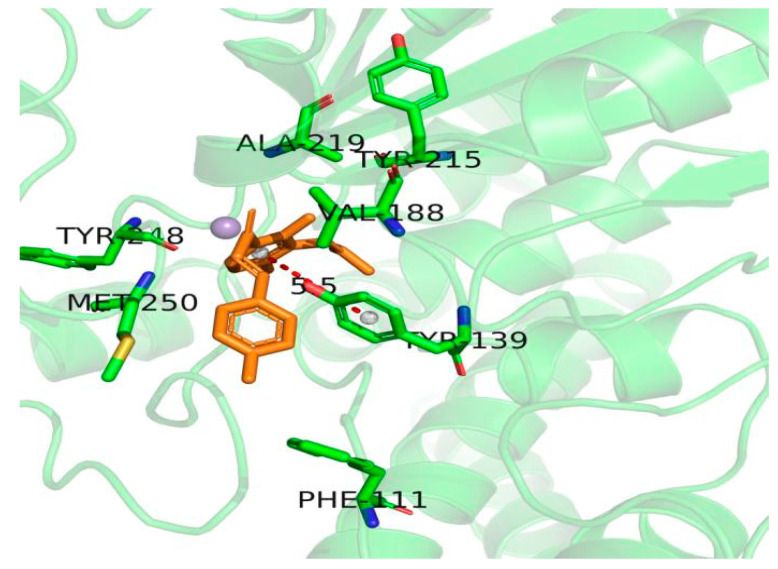
Pivotal interaction site of isobavachalcone and the pyruvate dehydrogenase of *M. oryzae.*

Molecular docking is a method for exploring the molecular interactions between molecules and receptors. There was no previous research to confirm the binding mode of IBC and pyruvate dehydrogenase E1, so the homology modeling structure of pyruvate dehydrogenase E1 was used as the target for the molecular docking study. It is necessary to understand which residues of pyruvate dehydrogenase are central in interactions. The analysis results showed no steric hindrance in the binding of IBC within the protein active pocket (Figure S3), indicating that IBC had a stable binding mode in the pyruvate dehydrogenase. The receptor-ligand interactions were divided into four types: hydrogen bonding, hydrophobic, water bridge, and ionic. IBC had π-π stacking interactions with residue TYR139 of pyruvate dehydrogenase E1; nonpolar amino acids, such as PHE111, VAL188, ALA219, and MET250, can form strong van der Waals interactions with IBC to significantly contribute to hydrophobicity (Figure S2 and Figure 7). In addition, most of the functional residues in docking belonged to the active site as determined by identifying conserved regions, which showed that the predicted result was consistent with the docking results. The results further demonstrate the reliability of the docking results.

### 2.6. Molecular Dynamics Simulation of the Pyruvate Dehydrogenase E1-IBC Complexes and Binding Free Energy

**Figure 8:**
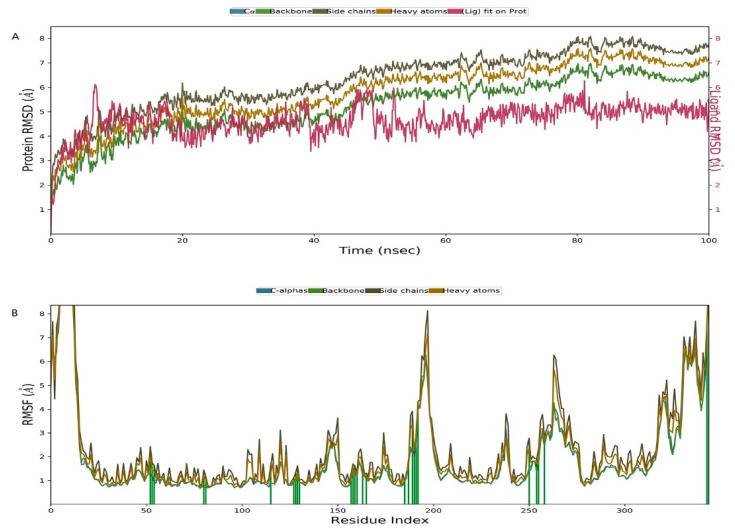
(**A**) RMSD of Cα atoms, backbone atoms, side-chain atoms, and heavy atoms in PDH-IBC complexes over time, during 100 ns MD simulations. (**B**) RMSF of backbone atoms, Cα atoms, side-chain atoms, and heavy atoms of PHD-IBC complexes during 100 ns MD simulations. A green vertical line marks protein residues interacting with IBC.

**Figure 9:**
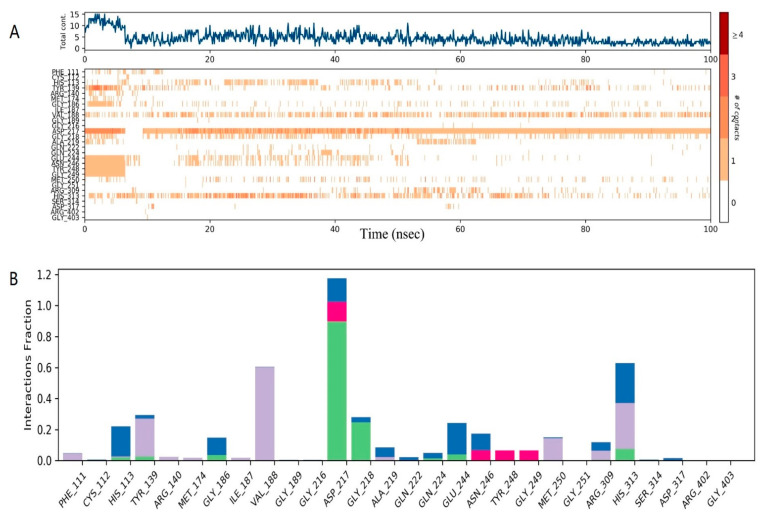
(**A**) During molecular dynamics, the residues interacted with the ligand in the framework of each trajectory. The top panel shows the total number of specific contacts the protein made with the ligand throughout the trajectory. Some residues made more than one specific contact with the ligand, which is represented by a darker shade of orange, according to the scale to the right of the plot. (**B**) Protein-ligand interactions can be divided into four types: hydrogen bonding, hydrophobic, ionic, and water bridge. Each interaction type contains more specific subtypes, which can be explored through the “Simulation Interactions Diagram” panel. The stacked bar charts were normalized during the trajectory. Values over 1.0 are possible as some protein residues may make multiple contacts of the same subtype with the ligand.

**Figure 10:**
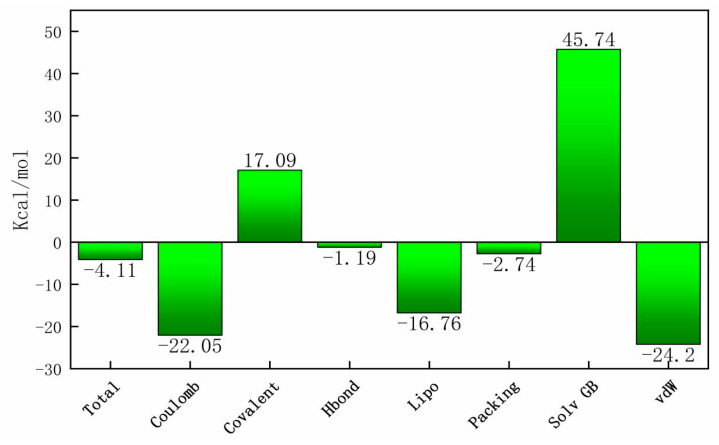
MM-GBSA binding energy of pyruvate dehydrogenase-IBC complexes.

Molecular dynamics simulation studies of ligand-protein complexes can monitor the interactions between docking complexes. The root-mean-square deviation (RMSD) is used to measure the average change in displacement of a selection of atoms for a particular frame concerning a reference frame, and the root-mean-square fluctuation (RMSF) is useful for characterizing local changes along the protein chain. The average RMSD values of heavy atoms of the complexes during MD simulation are shown in Figure 8A. Large fluctuations occurred initially, but the average RMSD of the protein increased linearly from 60 ns, then tended to be stable from 80 ns. Protein residues that interact with the IBC are marked with a green vertical line (Figure 8B). There were three main modes of interaction between pyruvate dehydrogenase E1 and IBC: hydrophobic, H-bonds, and water bridge interactions. For instance, ASP217 exhibited obvious H-bond interactions (89%, Figure 9). Furthermore, VAL188 and MET250 maintained highly hydrophobic interactions (100%), and other residues also maintained highly hydrophobic interactions, such as TYR139 (95%). Overall, the RMSD and RMSF analyses indicate that the complexes had certain conformational stability, which was due to the significant interactions between IBC and TYR139, VAL188, ASP217, GLY218, and MET250. Finally, we computed the MM-GBSA binding free energy of the pyruvate dehydrogenase E1-IBC complexes and the determined binding energy value was −4.11 kcal/mol (Figure 10).

## 3. Discussion

As one of the most destructive and widespread crop diseases, rice blast caused by *M. oryzae* has a significant negative impact on the production of rice [21]. Its management still relies on using fungicides. Due to the various problems caused by the long-term use of fungicides, it is essential to develop new modes of action or novel fungicidal agents on novel scaffolds [22]. Studies have shown that *Psoralea corylifolia* L. seed extract has antifungal activity against rice blast fungus; IBC is the major active component and has been widely used in the field.

In this paper, we explored the inhibition mechanism of IBC on *M. oryzae*. First, we found that the TCA cycle of *M. oryzae* was significantly affected at the transcriptome level following treatment with IBC. Genes corresponding to two important reductase systems of pyruvate dehydrogenase complexes and α-ketoglutarate dehydrogenase complexes, and pyruvate carboxylase, were selected for verification of changes in expression by qPCR, which revealed that pyruvate dehydrogenase E1 decreased most significantly after IBC treatment for 0.5 h. Moreover, pyruvate dehydrogenase E1 was extracted from the mycelium of *M. oryzae* after IBC treatment at 1, 2, 4, 8, and 16 h to clarify the inhibitory activity of IBC on pyruvate dehydrogenase E1. The results showed that the enzyme activity of pyruvate dehydrogenase E1 in the experimental group was obviously lower than in the control group. We speculate that pyruvate dehydrogenase E1 is a potential target that can be used against *M. oryzae*. PDHc is a target with important agronomic significance that is worth further exploring [19]. Studies have found that pyruvate dehydrogenase complex (PDHc) is the site of action of a new class of herbicides and algicide. For example, a series of α-(substituted-phenoxyacetoxy)-α-heterocyclyl methyl phosphonate derivatives (II) were synthesized as potential inhibitors of PDHc. The herbicidal activity was evaluated in a greenhouse with 15 weed species, and the activity of PDHc was determined by a sensitive spectrophotometric assay, which revealed that compounds II had significant herbicidal activity and caused effective inhibition of PDHc in plants [18,19]. Novel thiamin diphosphate (ThDP) analogs were designed and synthesized, targeting cyanobacterial pyruvate dehydrogenase complex E1, and showed inhibitory activities against Cy-PDHc E1 (IC50 9.56–3.48 µM) and inhibitory activities against two model cyanobacteria strains of *Synechocystis* sp. PCC6803 [23]. However, there have been few reports on developing fungicides targeting PDHc E1. In our study, the pyruvate dehydrogenase E1 of *M. oryzae* was selected as a potential target for further study of its binding mode with the fungicide IBC.

The essence of a drug’s efficacy results from the interaction between the small organic molecule of the drug and the receptor macromolecule after reaching its target point. Researching the interaction between drugs and target proteins at the atomic level often allows us to demonstrate the interaction mechanism. In this study, a 3D structure model of the pyruvate dehydrogenase E1 of *M. oryzae* was established based on homology modeling for the first time (Figure 4). Molecular docking and molecular dynamics were also utilized to explore molecular interactions and the binding mode between IBC and pyruvate dehydrogenase E1. The results showed that IBC had π-π stacking interactions with residue TYR139 and hydrogen bonding with residue ASP217 of pyruvate dehydrogenase E1. The interaction energies involving π systems are comparable in energy to hydrogen bonds and, therefore, contribute significantly to the binding free energy. Nonpolar residues PHE111, MET174, ILE 187, VAL188, and MET250, could form strong hydrophobic interactions, which increases the binding affinity of the ligand to its target. The analyses of molecular dynamics indicated that the complexes had certain conformational stability, which was due to the significant interactions between IBC and TYR139, VAL188, ASP217, GLY218 and MET250, which were consistent with the interactions of the docking study. These results indicate that IBC may occupy the active pocket of the pyruvate dehydrogenase E1 of *M. oryzae*. IBC binds to pyruvate dehydrogenase E1 through the force mentioned above and inhibits the activity of pyruvate dehydrogenase E1, thereby inhibiting the carbohydrate metabolism process of *M. oryzae*. As a result, the mycelium cells were gradually disrupted and died. We believe that pyruvate dehydrogenase E1 can be used as a potential target for which reasonable and effective fungicides could be further designed.

This study focused on the effect of IBC on the TCA cycle of *M. oryzae* at the transcriptome level. The binding mode of the target of PDHE1 and IBC was explored through homology modeling and molecular docking. However, some pathways were affected to varying degrees following IBC treatment for 2 h, such as ribosomes, the pentose phosphate pathway, glycolysis, etc., and there were significant changes in some differentially expressed genes that have not yet been verified. Additional transcriptomics data could be acquired in future research to discover new drug targets.

## 4. Materials and Methods

### 4.1. Fungal Strain

Strain MAFF 306679 of *M. oryzae* was isolated from diseased rice plants in Liaoning province, China, and was identified at Shanghai Majorbio Bio-pharm Technology Co., Ltd. (3399 Kangxin Road, Times Medical Innovation Park, Pudong New District, Shanghai, China).

### 4.2. Preparation of M. oryzae conidia

The mycelia of *M. oryzae* were grown on potato dextrose agar (PDA) plates at 28 °C for nearly two weeks, and conidia were induced under white fluorescence light for 72 h. The plate filled with mycelium was washed with 5 mL aseptic water, and the concentration was adjusted to 10^5^–10^7^ conidia/mL. Then, 5 mL conidia suspension was added to 150 mL liquid medium of PDA, and it was cultivated in thermostatic oscillation incubators at 28 °C with shaking at 150 rpm for five days.

### 4.3. Transcriptome Sequencing

The conidia suspension was divided into the experimental group and control group. Cultures in the experimental group were treated with 10 mg/L isobavachalcone (IBC, HPLC ≥ 98%, Pufei De Biotech Co, Ltd., Chengdu, China) for 2 h, with three replicates. An equivalent treatment with dimethyl sulfoxide (DMSO) was used for the control group. All samples were frozen in liquid nitrogen for two hours. Total RNA was isolated from the samples with a TRIzol Reagent kit (Invitrogen, CA) before RNA sequencing (RNA-Seq), which was utilized for cDNA library construction, sequencing, transcriptome assembly, sequence analysis and assembly, functional annotation, and classification. Differentially expressed genes (DEGs) between sample pairs were identified and filtered using R-packet DESeq2. Determination of DEGs between the two groups was based on |log_2_ (fold change)| ≥ 2 and padj < 0.05.

### 4.4. Detection by Quantitative Real-Time PCR

Eight genes were selected and analyzed by quantitative real-time PCR (qPCR) with normalization to a reference gene, actin, to validate the data of RNA-Seq. The conidia suspension of *M. oryzae* was prepared to obtain mycelium at 0.5, 1, 2, 4, and 8 h after treatment with 10 mg/L IBC. There were three replicates for all time points. The control group was treated with DMSO. RNA was isolated from each sample. The qPCR reaction was performed as follows: pre-denaturation at 95 °C for 30 s followed by 39 cycles of denaturation at 95 °C for 10 s, annealing at 60 °C for 20 s, and extension at 72 °C for 15 s. Data for real-time fluorescence was collected via detection during the annealing phase of each cycle, and the recorded point curves containing all specimens were obtained after the reaction. Quantification of the fold change of genes was analyzed using the Ct method [24].

### 4.5. Extraction of Solution for Enzyme Assays

Conidia suspension cultures developed for 5 days in the experimental groups were used to obtain mycelium at 1, 2, 4, 8, and 16 h after treatment with 10 mg/L IBC. All cultures had three replicates. Equal treatment of DMSO was used for the control group. After grinding into a powder, the mycelium was mixed with a 5 mL buffer solution of 50 mmol K_2_HPO_4_/KH_2_PO_4_ (pH 8.0), and the mixture was pulverized with an ultrasonic cell disruptor for half an hour. After being centrifuged for 15 min at 12,000 rpm, the supernatant was collected and stored at −80 °C.

### 4.6. Enzyme Activity Assays

The 2,6-DCPIP method was used to determine the activity of the pyruvate dehydrogenase E1 component [25]. The preparation of the standard curve is shown in Table S1. The reaction medium included (in a 3 mL test volume) 0.6 mL buffer solution of 50 mmol K_2_HPO_4_/KH_2_PO_4_ (pH 7.1), 0.3 mL 1.0 mmol L^−1^ MgCl_2_, 0.3 mL 0.2 mmol L^−1^ TPP, 0.1 mL 0.1 mmol L^−1^ DCPIP, 0.3 mL 2.0 mmol L^−1^ sodium pyruvate, and 1.3 mL triple steamed water. The reaction was first heated in a water bath for 5 min at 30 °C. Then, a 0.1 mL enzyme solution was added to initiate the reaction. The absorbance was continuously measured over a 5 min period at OD_600_, and the control group, without DCPIP, was used as the reference sample. The activity of the enzyme was calculated by the following formula. One unit of pyruvate dehydrogenase E1 component activity is defined as the amount of 1 μmol 2,6-DCPIP consumed per mg of tissue per minute (μmol 2,6-DCPIP·mg^−1^·pro·min^−1^) [25,26]:

U = ((Ax − A0) × Vt)/(FW × Vx × d × t)

Ax − A0: The amount of 2,6-DCPIP, which is reduced; Vt: total reaction volume (3 mL); FW: mycelium fresh weight; Vx: enzyme volume (0.1 mL); d: Petri dish diameter (1 cm).

### 4.7. Preparation of the Ligand Structure

The molecular structure of the ligand was constructed using Marvin Sketch (ChemAxon, Hungary), as shown in Figure 4A.

### 4.8. Homology Modeling

The 3D structure of pyruvate dehydrogenase E1 was modeled using the SWISS-MODEL online server (https://swissmodel.expasy.org/, accessed on 17 December 2020). The protein sequence of the pyruvate dehydrogenase E1 component from *M. oryzae* was retrieved from the UniProt database (https://www.uniprot.org/, accessed on 17 December 2020), with accession number G4N7T0 [27]. The search was performed to find a suitable protein template of known 3D structure [28]. We found that the best match for the protein sequence of the pyruvate dehydrogenase E1 component was the crystal structure of the pyruvate dehydrogenase (E1p) component of the human pyruvate dehydrogenase complex (PDB code: 3exe), with a sequence identity of 55.13%. Based on this, the SWISS-MODEL online server comparatively modeled the 3D protein structure of the pyruvate dehydrogenase E1 component [29], automatically generating an energy-minimized protein model and satisfying the space constraints of the bond distance and dihedral angles [30]. The established 3D structure was finally evaluated by the Procheck and Ramachandran Plot analysis [31].

### 4.9. Identification of Pyruvate Dehydrogenase Active Site

The residues that play an important role in maintaining protein function are highly conserved and, hence, identifying extremely conserved regions of a protein sequence can help to determine functionally important residues. The ConSurf online server (http://consurf.tau.ac.il/, accessed on 17 December 2020) was used to predict the theoretically active site of the pyruvate dehydrogenase E1 component receptor [32]. Considering the phylogenetic relationship between proteins, the functional regions in the protein were quantified according to the conservation status of the amino acid sequence [33].

### 4.10. Molecular Docking Studies

In structural molecular biology and computer-assisted drug design, molecular docking is a key computer-based technique, which is used to investigate the predominant binding mode between a ligand and a protein of known 3D structure or, in other words, a receptor [34,35]. To explore the interaction mechanism and potential binding sites of IBC with pyruvate dehydrogenase E1, the molecular docking between IBC and the homology model of pyruvate dehydrogenase E1 were determined by using glide software, which is a module of Maestro/Schrödinger’s molecular modeling software package [36]. The active sites of the 3D structure of the pyruvate dehydrogenase protein were predicted using Discovery Studio client 16 software (Accelrys, San Diego, CA, USA) [37], which found there were nine active sites where docking was carried out. Finally, conformations were obtained and ranked by glide score, which is an empirical scoring function that combines multiple parameters (Table S2) [38], The unit of the glide score was kcal/mol, including ligand-receptor interaction energies, π-π stacking interactions, hydrogen bonding terms, hydrophobic interactions, desolvation, and root-mean-square deviation (RMSD) [38]. The optimal active site was selected based on the glide score for further analysis.

### 4.11. Molecular Dynamics Simulations

Molecular dynamics (MD) simulation of the ligand-protein binding of the pyruvate dehydrogenase protein and IBC was performed using the Desmond v3.8 modules in Schrödinger suit. In the MD simulation, an appropriate amount of Na^+^ counter-ions were used to neutralize the charges in the complexes. To evaluate the stability of the pyruvate dehydrogenase E1-IBC complexes, the complexes were first minimized and subjected to MD in the NVT ensemble. The temperature of the system was raised from 0 to 300 K. After the initial equilibration. A molecular dynamics production run was carried out for 100 ns. Different parameters of the MD simulation study were investigated, such as analysis of the ligand-binding site, RMSD, and protein root mean square fluctuation (RMSF), etc. [39].

### 4.12. Binding Free Energy Calculation

The molecular mechanics-generalized Born surface area (MM-GBSA) method was used to calculate the binding free energies of pyruvate dehydrogenase E1-IBC complexes, using the Schrödinger software suite.

## 5. Conclusions

In our previous studies, the inhibition mechanism of IBC of *M. oryzae* focused on the physiological and biochemical level, which clarified that IBC could activate the activity of chitin hydrolase to destroy the cell wall structure. In our study here, we explained the inhibition mechanism of IBC on *M. oryzae* at the molecular level using a transcriptomics platform, which is very important for identifying the target of a drug. This research found that the TCA cycle of *M. oryzae* was affected after IBC treatment for 2 h by transcriptome sequencing; the expression of its key enzyme, pyruvate dehydrogenase, was significantly downregulated. Pyruvate dehydrogenase E1 was then extracted from *M. oryzae*, and an experiment on enzyme activity was carried out to identify the inhibitory activity of IBC on the pyruvate dehydrogenase E1 of *M. oryzae*. The activity of pyruvate dehydrogenase in the experimental group at 1, 2, 4, 8, and 16 h was 0.87, 0.8, 0.74, 0.62, and 0.45 times as much as in the control group respectively. Hence, the results suggest pyruvate dehydrogenase E1 may be a potential antifungal target.

To further illuminate the interaction mechanism between IBC and pyruvate dehydrogenase E1, the 3D structure of pyruvate dehydrogenase E1 in *M. oryzae*—based on the principle of homology modeling—was established for the first time, and the model was verified by Procheck. Molecular docking was then carried out to explore the molecular interactions and elucidate a model for binding between IBC and pyruvate dehydrogenase E1. The molecular docking results demonstrated that the interactions between pyruvate dehydrogenase E1 and IBC resulted from H-bonding, π-π stacking, and strong van der Waals interactions involving TYR139, VAL188, ASP217, GLY218, and MET250. In addition, molecular dynamics analysis was performed on the stable structure of the pyruvate dehydrogenase E1-IBC complex. RMSF and RMSD analyses indicated that the molecular system had certain conformational stability. The binding energy value of the complexes was calculated as −4.11 kcal/mol.

The above research may be useful for designing new, effective fungicides targeting pyruvate dehydrogenase E1. By analyzing the binding site of the target-ligand complexes, a ligand could be constructed according to the active site of the target, and a compound with biological activity could then be designed. We expect we will discover new targets by studying other metabolic pathways in the future.

## Figures and Tables

**Table 1:** Genes in the pathway of the TCA cycle.

Gene	Definition	FC (C/B)	|Log_2_ (FC)|
4.1.1.49	Phosphate carboxykinase (ATP)	0.126	2.989
1.2.4.1	Pyruvate dehydrogenase E1 component	0.139	2.847
2.3.2.12	Pyruvate dehydrogenase E2 component (dihydrolipoamide acetyltransferase)	0.189	2.404
1.8.1.4	Dihydrolipoamide dehydrogenase	0.126	2.989
6.4.1.1	Pyruvate carboxylase	0.069	3.857
2.3.3.1	Citrate synthase	0.071	3.816
2.3.3.8	ATP citrate (pro-S)-lyase	0.086	3.540
4.2.1.3	Aconitate hydratase	0.187	2.419
1.1.1.41	Isocitrate dehydrogenase (NAD+)	-	-
1.1.1.42	Isocitrate dehydrogenase	-	-
1.2.4.2	2-Oxoglutarate dehydrogenase E1 component	0.105	3.252
2.3.1.61	2-Oxoglutarate dehydrogenase E2 component (dihydrolipoamide succinyltransferase)	0.154	2.699
4.2.1.2	Fumarate hydratase, class I	0.144	2.786
1.3.5.1	Succinate dehydrogenase (ubiquinone) flavoprotein subunit	0.149	2.796
6.2.1.4	Succinyl-CoA synthetase alpha subunit	0.119	3.071
6.2.1.5	Succinyl-CoA synthetase alpha subunit	0.119	3.071

Genes marked with blue and red borders in Figure 1 are those whose expression levels have changed. FC(C/B) denotes the differential expression of this UniGene between two samples, and B is the control group. Log_2_FC is the fold change value of the expression difference between the two groups. The smaller the FC value and the larger the |Log_2_(FC)| value, the more obvious the gene downregulation. The genes marked in red are those verified by qPCR.

## Data Availability

Not applicable.

## Supplementary Materials

The following are available online at https://www.mdpi.com/article/10.3390/ijms22105163/s1. Figure S1. Sequence alignment between the pyruvate dehydrogenase query sequence and the template amino acid sequence.
