# Supplementary material for: Identification of Pyruvate Dehydrogenase E1 as a Potential Target against Magnaporthe oryzae through Experimental and Theoretical Investigation
Source: Int J Mol Sci. 2021 May 13;22(10):5163. doi: 10.3390/ijms22105163 (PMC8153330; doi:10.3390/ijms22105163)
Supplement: Supplementary file 1 [file ijms-22-05163-s001.zip › ijms-1201210-supplementary.pdf]

Supplementary materials

Table S1: Production of standard curve.

The number of tube	1	2	3	4	5	6
10*0.1mM DCPIP(μL)	0	37.5	75	150	225	300
Distilled water (μL)	300	262.5	225	150	75	0
10*1mmol^{-1} $MgCl_2$ (μL)	300	300	300	300	300	300
10*0.2mmol^{-1} TPP (μL)	300	300	300	300	300	300
10*2.0mmol^{-1} acetone Acid (μL)	300	300	300	300	300	300
Buffer solution of 10*50mmol K_2HPO_4&KH_2PO_4 (pH7.1) (μL)	600	600	600	600	600	600
Distilled water (μL)	1200	1200	1200	1200	1200	1200
Concentration of DCPIP solution (μM)	0	12.5	25	50	75	100
Absorbance values(OD_{600})	0	0.177	0.356	0.698	1.005	1.336

Table S2: The results of molecular docking. The active site of the pyruvate dehydrogenase model were predicted by Discovery Studio client 16 software. There are 9 active sites and one by one for molecular docking. Then ranked by glide gscore which is an empirical scoring function that combines multiple parameters. There was no docking result at site 6, so delete it.

cite	RMS Derivative-OPLS 2005	RMS Derivative-OPLS 2005	Docking score	XP GScore	Glide gscore	Glide energy	XP HBond	XP PoseRank
Cite1	0.047	0.020	-5.008	-5.038	-5.038	-30.813	-1.180	1
Cite2	0.047	0.020	-4.930	-4.959	-4.959	-34.133	-0.699	1
Cite3	0.047	0.020	-2.614	-2.644	-2.644	-31.786	-0.787	1
Cite4	0.047	0.020	-3.950	-3.980	-3.980	-32.925	-0.998	1
Cite5	0.047	0.020	-3.084	-3.114	-3.114	-34.163	-1.131	1
Cite7	0.047	0.020	-3.662	-3.692	-3.692	-35.447	-0.975	1
Cite8	0.047	0.020	-3.693	-3.723	-3.723	-33.247	-1.003	1
Cite9	0.047	0.020	-3.887	-3.917	-3.917	-34.285	-1.002	1

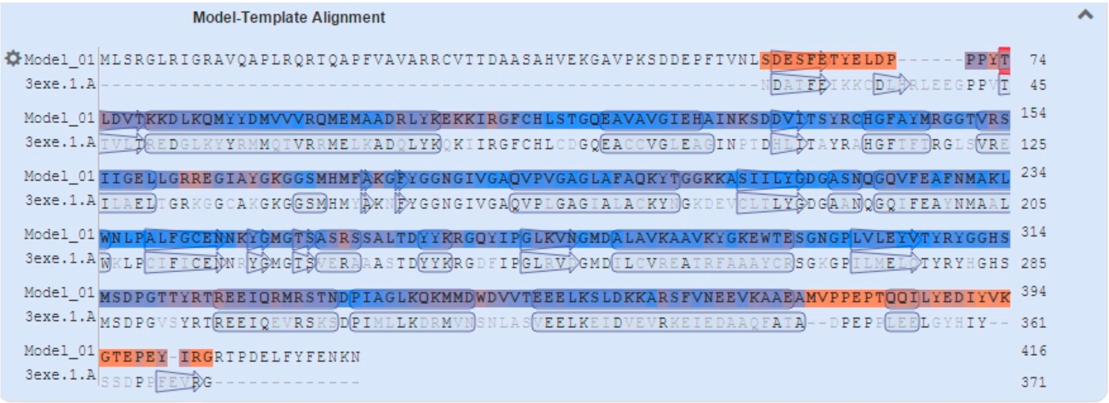

Figure S1: Sequence alignment between the pyruvate dehydrogenase query sequence and the template amino acid sequence.

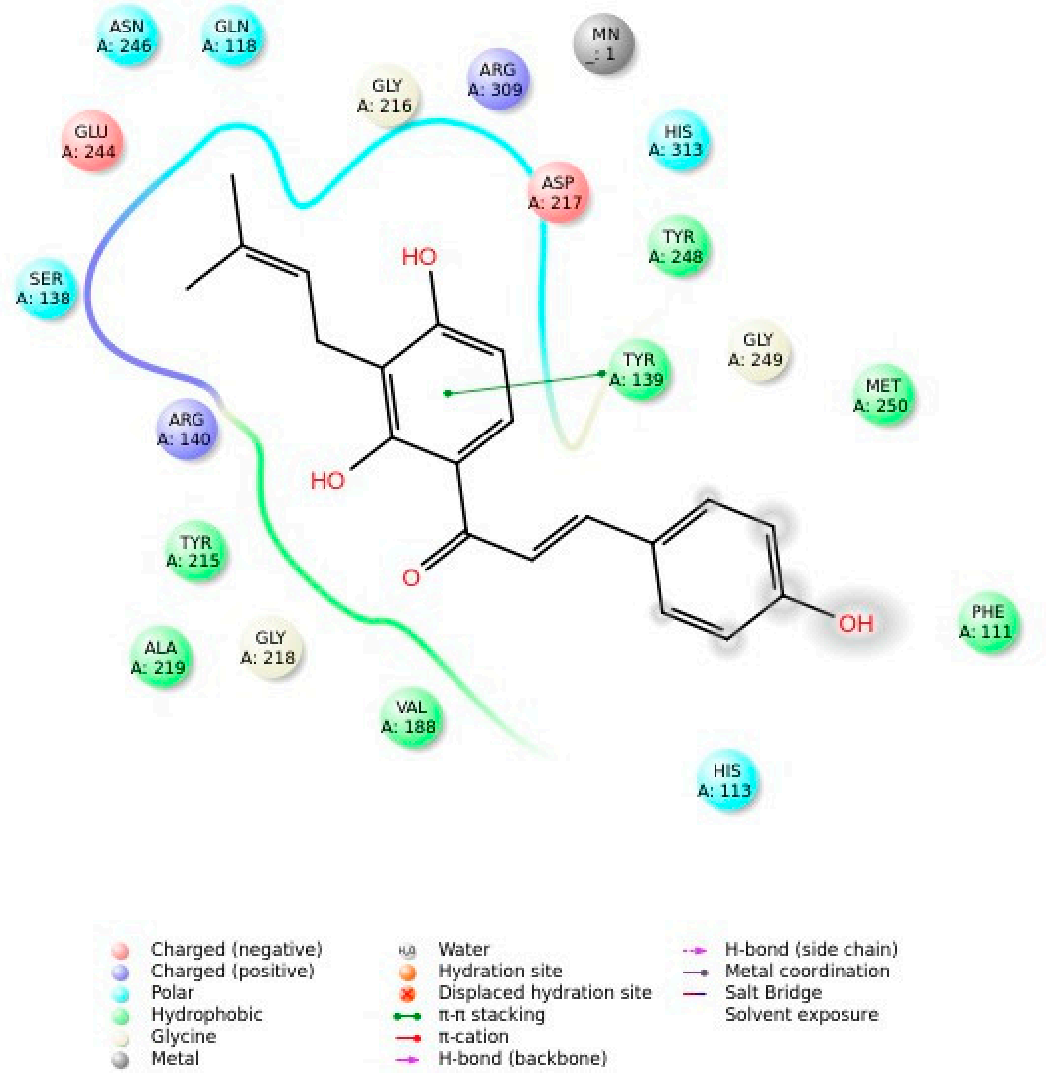

Figure S2. The pivotal interaction site of isobavachalcone and the pyruvate dehydrogenase of *M. oryzae*.

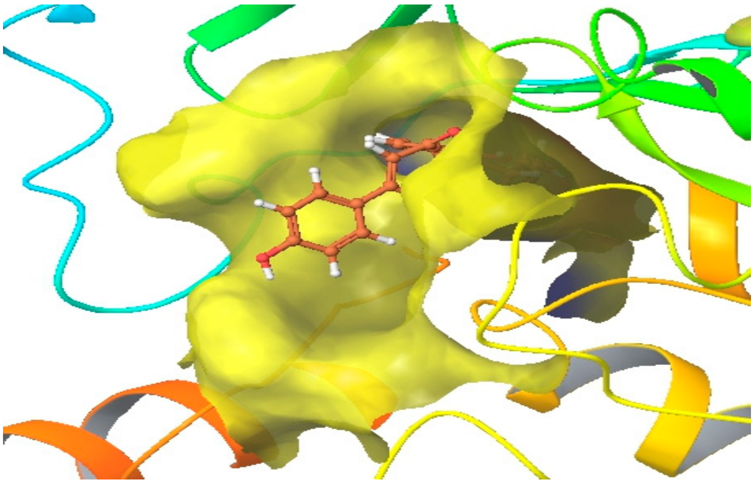

Figure S3. The binding mode in the active pocket of IBC and pyruvate dehydrogenase. The compound is well wrapped.
